# NEIGHBORHOOD SOCIOECONOMIC STATUS AND CHANGES IN COGNITIVE FUNCTION IN OLDER ADULTS: FINDINGS FROM HEALTH ABC STUDY

**DOI:** 10.1093/geroni/igad104.1187

**Published:** 2023-12-21

**Authors:** Greta Jianjia Cheng, Sara Godina, Erica Fan, Andrea Rosso

**Affiliations:** University of Pittsburgh, Pittsbugrh, Pennsylvania, United States; University of Pittsburgh School of Public Health, Pittsburgh, Pennsylvania, United States; University of Pittsburgh, School of Public Health, Pittsburgh, Pennsylvania, United States; University of Pittsburgh, Pittsburgh, Pennsylvania, United States

## Abstract

Neighborhood socioeconomic status (NSES), an overall marker of neighborhood condition, may influence multiple aspects of health and well-being in older adulthood, including cognitive function, though results regarding neighborhood and cognition have been largely cross-sectional. We examined the association between NSES and changes in cognitive function using 610 participants from the Pittsburgh site of the Health, Aging, and Body Composition (Health ABC) Study, a longitudinal cohort of community-dwelling, Black and white adults aged 70 and older. Changes in cognitive function were measured annually over a 6-year follow-up (2006-2012) using the Modified Mini-Mental State Examination (range: 0-100, 3 visits on average per person). Using baseline home address, NSES was calculated for census tracts by summing z-scores of six 2010 census variables representing wealth/income, education, and occupation, and divided into tertiles. Multivariate mixed-effect linear regression models assessed associations, adjusting for individual-level sociodemographic and health-related covariates. In the fully adjusted model, the lowest NSES tertile (vs. the highest) was not associated with baseline cognitive function (β: -0.99, 95% CI: -3.86, 1.87) but was associated with a faster rate of decline in cognitive function (β: -1.54, 95% CI: -2.67, -0.41). No significant differences in baseline cognitive function or rates of change were observed in the middle (vs. the highest) NSES tertile. There was no evidence of effect modification by race. The results indicate that exposure to adverse neighborhood socioeconomic environments may accelerate cognitive decline in late life. The findings add to existing literature concerning the role of environments in the context of aging.

